# PROMIS COGNITIVE FUNCTION AND CONCERN SCALES: LINGUISTIC VALIDATION IN AMERICAN SIGN LANGUAGE FOR DEAF OLDER ADULTS

**DOI:** 10.1093/geroni/igz038.2880

**Published:** 2019-11-08

**Authors:** Poorna Kushalnagar

**Affiliations:** Gallaudet University, Washington, District of Columbia, United States

## Abstract

Objective: To culturally and linguistically validate PROMIS Cognitive Functions and Concerns Scales in American Sign Language (PROMIS-ASL) for use with deaf older adults over 50. Methods: We used the standard procedures developed at the U.S. National Center for Health Statistics Cognitive Survey Laboratory to culturally adapt and translate items from the cognitive scales of the Patient Reported Outcomes Measurement Information System (PROMIS). We describe cultural adaptation and linguistic translation procedures led by a team of primarily deaf investigators. Using multidimensional exploratory or confirmatory factor analyses, we identify or confirm the items most likely to comprise each subset. Once the PROMIS-ASL version is finalized, we will compute test-retest reliability using ICC (intraclass correlation coefficient) from two-way random effects ANOVA models. Results: We produced an accessible patient reported outcomes cognitive measure in American Sign Language and a culturally appropriate set of items that are relevant to the experiences of deaf older adult over 50 users of accessible technology and services. Conclusions: The final PROMIS-ASL product with cognitive domain will be distributed for public use.

